# INTERNET UTILIZATION AMONG OLDER ADULTS WITH VISUAL IMPAIRMENT

**DOI:** 10.1093/geroni/igae098.0114

**Published:** 2024-12-31

**Authors:** Rachel Mumby, Philippa Clarke, Yunshu Zhou, David Rein, Joshua Ehrlich

**Affiliations:** University of Michigan, Lansing, Michigan, United States; University of Michigan, Ann Arbor, Michigan, United States; University of Michigan, Ann Arbor, Michigan, United States; NORC at the University of Chicago, Chicago, Illinois, United States; University of Michigan, Ann Arbor, Michigan, United States

## Abstract

Internet is increasingly used by older adults for communication, socialization, household tasks, and health information/care. Although the internet is most often accessed by visual means, little is known about whether vision impairment is associated with internet use in older adults. To address this question, we used nationally representative data from round 12 (collected in 2022) of the National Health and Aging Trends Study (NHATS), which included measures of visual function (distance visual acuity, near visual acuity, contrast sensitivity) and a series of questions about computer and internet use among US adults aged 65 and older. Data were analyzed using survey weighted multivariable logistic models adjusted for socioeconomic, demographic, and health-related covariates. There were 5,220 participants included (weighted: 45.6% male, 79.9% non-Hispanic White). Among those with any type of vision impairment, there were significantly lower odds of internet use (OR=0.59, 95% CI 0.47-0.73), email and texting (OR=0.52, 95% CI 0.40-0.68), online household activities (e.g., banking, grocery shopping; OR=0.69, 95% CI 0.57-0.84), online social networking (OR=0.64), 95% CI 0.52-0.80), and online health activities (e.g., prescription refills, telehealth; OR=0.64, 95% CI 0.50-0.82) compared to those without any vision impairment. Most of these associations were driven by near vision impairment. Although internet use has increased among older adults overall, there is a gap in utilization between older adults based on vision impairment status. Findings suggest a need for interventions to make the internet more accessible to older adults with impaired vision.

